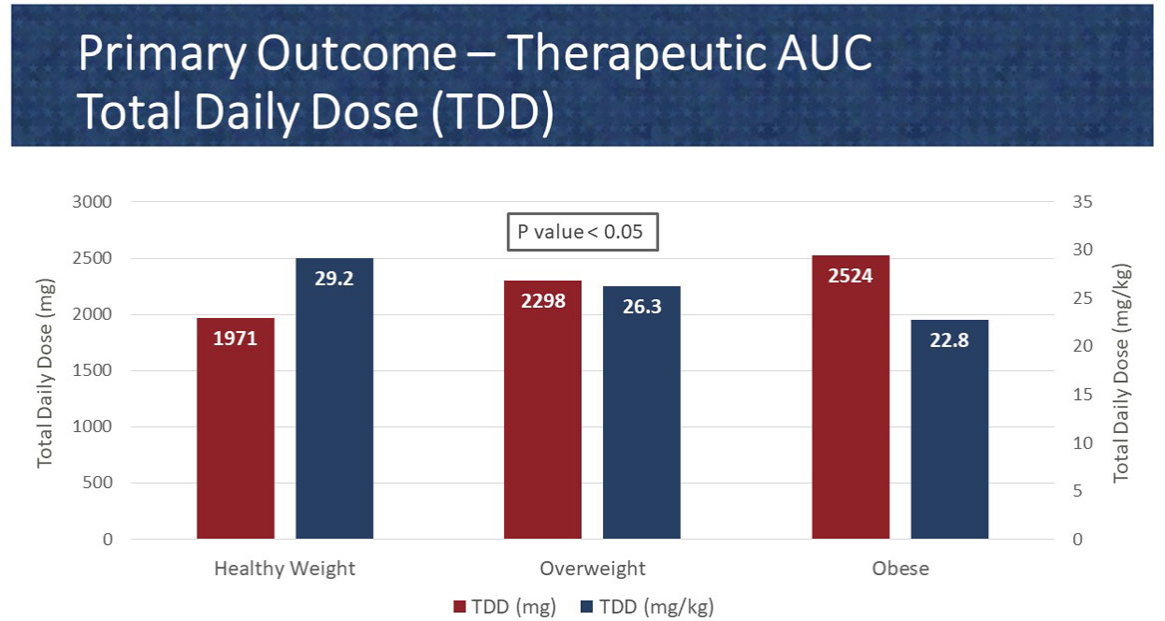# Optimal Weight-Based Dosing of Vancomycin to Achieve an Area Under the Curve of 400 to 600 Stratified by Body Mass Index

**DOI:** 10.1017/ash.2024.296

**Published:** 2024-09-16

**Authors:** Caroline Williams, Anna Mitchell, Jessica Bennett, Ally Ponder

**Affiliations:** Lt. Col. Luke J. Weathers, Jr. VA Medical Center; Department of Veterans Affairs

## Abstract

**Background:** In 2020, the American Society of Health System Pharmacist (ASHP) and Infectious Diseases Society of America (IDSA) published a consensus guideline for vancomycin management, recommending area under the curve (AUC) as the preferred monitoring strategy. These guidelines recommend doses of 15-20 mg/kg every 8 to 12 hours for most patients with normal renal function. However, in extreme body weights, standard dosing may deviate to provide a therapeutic AUC. The primary objective of this pharmacokinetic study is to evaluate the optimal vancomycin weight-based dosing strategy that achieves a therapeutic AUC of 400-600 stratified by body mass index (BMI). The secondary objective is to evaluate the incidence of acute kidney injury (AKI) based on BMI. **Methods:** Patients were identified from two sites within the Department of Veterans Affairs who received vancomycin for at least 48 hours and had at least one steady-state level from January 2015 through July 2022. Regimens with a frequency of ≤8 hours or patients with baseline creatine clearance of < 5 0 ml/min were excluded. Patients were categorized based on the Center for Disease Control BMI groups: healthy weight, overweight, or obese. The online vancomycin calculator, VancoPK©, was utilized to calculate AUC. Renal function at baseline and during vancomycin therapy was collected. Descriptive statistics were used for data analysis. Continuous outcomes were summarized using mean and standard deviation. The primary and secondary endpoints were analyzed using the analysis of variance and Fisher’s exact tests, respectively. Statistical significance was established at a p-value of < 0 .05. **Results:** A total of 347 unique vancomycin regimens were included: 120 in the healthy weight group, 101 in the overweight group, and 126 in the obese group. The average total daily doses that achieved a therapeutic AUC were 1971mg (15.6mg/kg/dose), 2298mg (15mg/kg/dose), and 2524mg (12.6mg/kg/dose) for the healthy weight, overweight, and obese groups, respectively. There was a statistically significant difference among these groups. AKI occurred in 10/254 (3.9%) unique patients: 2/89 (2.2%) in the healthy weight group, 3/71 (4.2%) in the overweight group, and 5/94 (5.3%) in the obese group. This did not reach statistical significance. **Conclusions:** Vancomycin dosing regimens largely followed guideline recommendations. However, the average vancomycin mg/kg/dose that achieved a therapeutic AUC decreased as BMI increased, which was a statistically significant trend. While further research is needed to draw clinically impactful conclusions, these findings suggest that a lower mg/kg vancomycin dose in obesity may be needed to achieve therapeutic targets.

**Figure:**
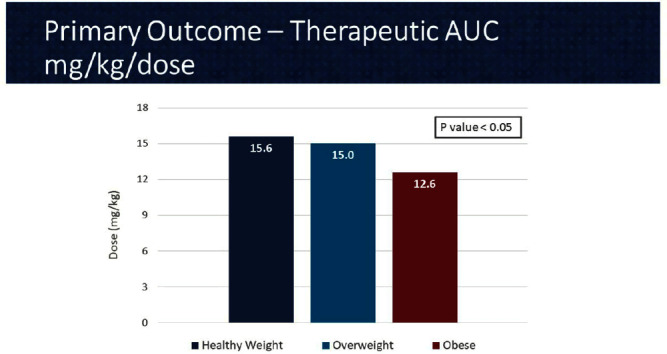

**Figure:**